# Magpie Trial in the UK: methods and additional data for women and children at 2 years following pregnancy complicated by pre-eclampsia

**DOI:** 10.1186/1471-2393-9-15

**Published:** 2009-04-14

**Authors:** Rebecca MD Smyth, Patsy Spark, Nina Armstrong, Lelia Duley

**Affiliations:** 1 Division of Public Health, University of Liverpool, UK; 2 National Perinatal Epidemiology Unit, University of Oxford, UK; 3 Centre for Epidemiology and Biostatistics, University of Leeds, UK

## Abstract

**Background:**

The Magpie Trial, a randomised trial comparing magnesium sulphate with placebo for women with pre-eclampsia. This paper describes methods used for follow up in the UK, and presents additional data collected.

**Methods:**

In the UK 774 women and their 827 children were included; excluded were women discharged without a surviving child and families who opted out. General practitioners were sent a questionnaire when the child was around 18 months old. When the child was two years, or older, questionnaires asking about the health of the women and children were posted to families. A sample of families was offered a home visit, during which the child was assessed using the Bayley Scales of Infant Development.

**Results:**

Of the women, 12 were lost to follow up and three died. Of the children, 12 were lost to follow up, 5 were excluded and 19 died. General practitioners returned 688/759 (91%) questionnaires, as did 619/759 (82%) women. Responses were largely comparable. 32 women had serious morbidity potentially related to pre-eclampsia. 30% of children were reported to have been admitted to hospital. There were no clear differences between the randomised groups in the child's behaviour, women's fertility or use of health service resources.

**Conclusion:**

Data presented here provide further reassurance about the longer term safety of magnesium sulphate when used for women with pre-eclampsia. Postal questionnaires in the UK to assess the longer term health and wellbeing of women and children recruited to trials are feasible, and can achieve a high response rate. Responses from families and general practitioners were comparable

**Trial registration:**

Trial registration number of the Magpie Trial [ISRCTN86938761]

## Background

Pre-eclampsia; a multisystem disorder of pregnancy usually associated with raised blood pressure and proteinuria, complicates 2–8% of pregnancies[1]. Although outcome is generally good, pre-eclampsia is a major cause of morbidity and mortality for the woman and her child [2]. For example, it accounts for an estimated one fifth of antenatal admissions[3], two thirds of referrals to day care assessment units[4] , and a quarter of obstetric admissions to intensive care units[5]. Eclampsia is defined as the occurrence of one or more convulsions superimposed on pre-eclampsia. Fortunately in the UK eclampsia is rare, affecting around 1 in 2000 deliveries, although it is associated with a high morbidity and mortality [6]. Pre-eclampsia and eclampsia together account for 15% of direct maternal deaths in the UK, with two-thirds related solely to pre-eclampsia [7].

The Magpie Trial compared magnesium sulphate with placebo for women with pre-eclampsia[8]. This randomised trial demonstrated that magnesium sulphate more than halves the risk of eclampsia (relative risk (RR) 0.42, 95% confidence interval (CI) 0.29–0.60), and probably reduces the risk of maternal death before discharge from hospital (RR 0.55, 95% CI 0.26–1.14). No substantive harmful effects were apparent for the mother or baby, in the short term.[8] Follow up at 18 months, or later, found little evidence that *in utero *exposure to magnesium sulphate is associated with an increase in the risk of death or neurosensory disability for the child [9] , or in maternal death or serious morbidity potentially related to pre-eclampsia for the mother [10].

For the follow up study; methods for tracing and assessing families were developed that could be implemented across 19 countries and a range of settings.[11,12] Nevertheless, in the UK methods of follow up differed from other countries in several ways; deaths were ascertained through the national registration system; tracing and assessment of UK women and children was co-ordinated centrally, rather than through local co-ordinators; screening was by postal questionnaires and this included both general practitioner and the family; and some additional questions were used in the UK, but not in other countries. This paper describes the methods used in the UK and presents data collected only in the UK, which have not been published previously.

## Methods

**Figure 1:**
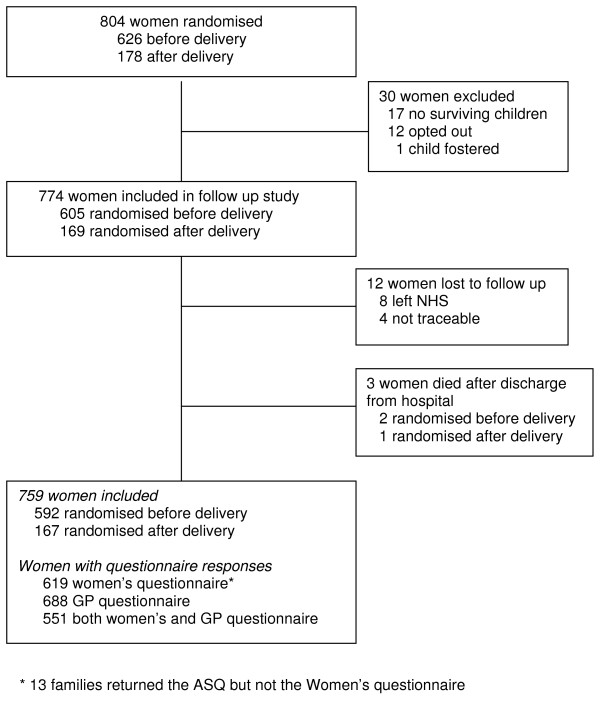
**Profile of women recruited in the UK**.

**Figure 2:**
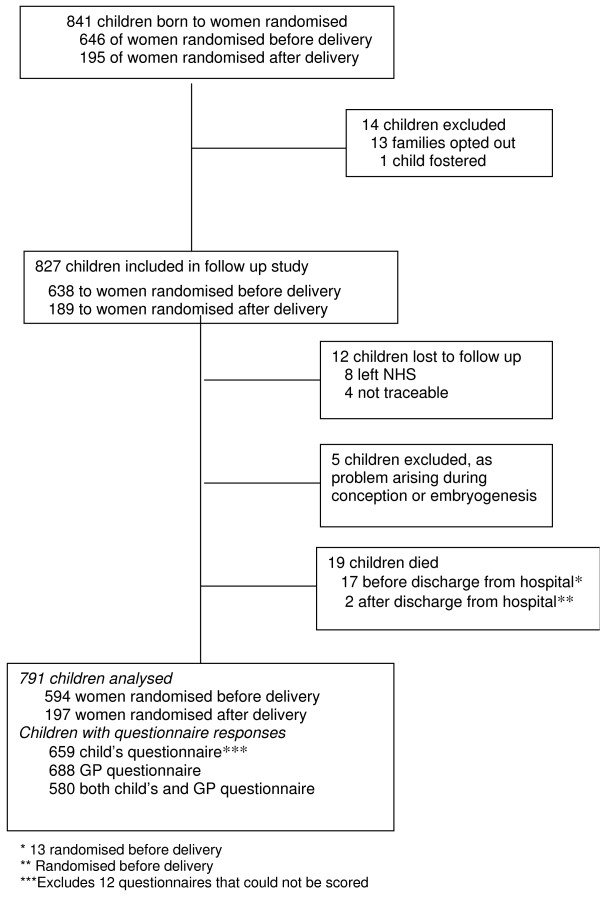
**Profile of children born to women recruited in the UK**.

Between July 1998 and November 2001, 804 women were recruited to the Magpie Trial at 67 UK hospitals. Follow up was from October 2002 until May 2004. Thirty women were excluded from tracing leaving 774 eligible for follow up (Figure 1). Overall, 841 children were born to women recruited, of whom 14 were excluded (Figure 2) leaving 827 eligible for follow up. Of these, 638 children were born to women randomised before delivery. Women and children who died after discharge from hospital were included in the overall analysis of outcome [9,10] but as the focus of this paper is the questionnaires, these deaths are excluded here.

Ethics approval was from the Northwest Multicentre Research Ethics Committee, as an amendment to the original trial protocol. Women had been informed they might be contacted for follow up before agreeing to recruitment, so additional consent was not required. All women and carers could opt out of the study at any time, if they wished.

### How families were traced

Ascertainment of deaths after discharge from hospital was through the Office of National Statistics. Families were contacted if there was at least one surviving child. If the mother had died, the child's carer was contacted.

The families' current address was obtained from a range of sources, including trial data collected at discharge from hospital, the Office for National Statistics, and the National Health Service Tracing Scheme. We wrote to each woman describing the follow up study, enclosing a simple card asking her to let us know if she changed address, and giving her the opportunity to 'opt out'. This letter was sent with either a birthday card for the child's first birthday, or a greetings card.

### How the children and women were assessed

The tools for screening and assessment of the children and women are described in our protocol. [11] In the UK we first sent a questionnaire to the general practitioner when the child was 18 months old. This was also to check that it was appropriate to contact the family. If the woman and child had different general practitioners, both were contacted. The general practitioner questionnaire included questions about the child's general health since birth, recent consultations (excluding routine assessments and immunisations), neurosensory function, any diagnosis, prescribed medication, and admission to hospital. A separate section asked about the mothers' health, possible long-term sequelae of pre-eclampsia, prescribed medication, and admission to hospital. Those who did not respond were sent reminders, or contacted by telephone.

When children were 2 years old, or more, the child's questionnaire was posted to the family, along with a second questionnaire asking the mother about her health. Reminders were sent after one month. If there was no response, a second reminder was sent by recorded delivery, or the family was contacted by telephone.

The child's questionnaire incorporated the Ages and Stages Questionnaires (ASQ)[13] with some additional questions about the child's behaviour and use of health care resources:

#### Behaviour

1) Does your child play happily with toys for up to 10 minutes?

2) Is your child frightened in new situations?

3) Does your child play happily with other children if you are around?

4) Does your child cling to you when you are with other people?

5) Does your child settle easily to sleep throughout the night?

*Options for response to questions '*Yes' 'Sometimes' 'No'

#### Use of health care resources

6) Has your child been prescribed any medicines to be taken for more than two weeks?

If yes, please give their names ..................................................................

7) Over the last 3 – 6 months have you taken your child to the general practitioner?

If yes, how many times and what for ............................................................

The accompanying women's questionnaire asked women about their own health, again with additional questions used only in the UK:

#### Fertility

1) Have you used contraception since your child was born?

If yes which ones ................................................................

2) Did having pre-eclampsia (toxaemia) contribute to your choice?

If yes, please explain how ...........................................................

3) Have you tried to get pregnant again since your child was born?

If no, did having pre-eclampsia contribute to your decision?

Please explain ...................................................................

#### Use of health care resources

4) Have you seen anyone else about your health, or how you feel, since your child was born?

If yes, who and what for? .......................................

*Options for response to questions '*Yes' or 'No'

Women were also asked about their experience of participating in the Magpie Trial. Responses to these questions are part of a qualitative analysis of participants' views of being in the trial, and are reported elsewhere [14].

##### Further assessment of children and women

Children were considered 'screen positive' if they failed the ASQ, if the ASQ could not be scored, or if the child passed an ASQ for a younger age group. The families of these children were contacted by telephone and offered a home visit for further assessment. Children were considered screen negative if they passed the ASQ for their own age group, or for an older child. Screen negative children in the north east of England were offered a home visit.

The aim of the home visit was to confirm whether or not the child had neurodevelopmental delay, or any other significant problem. If so, to collect information that would, if possible, establish a diagnosis. Families were asked about the child's current health and development, and the child was tested using the Bayley Scales of Infant Development (BSID-II) [15]. These assessments were conducted by a midwife and a psychologist, both trained in use of the BSID-II, who met every three months and conducted some joint visits.

During the home visit we took the opportunity to measure the woman's blood pressure, and to ask her about her health. Home visits lasted approximately two to three hours. All assessments were blind to the allocated treatment.

### Statistical analyses

Responses by the general practitioner and the family were described and compared for both women and children. As information was collected from general practitioners earlier than from families, the aim was to assess whether these responses were comparable and whether differences could reasonably be expected to be due to the time difference. Data assessing the child's behaviour, the woman's fertility, and their use of health service resources were compared between the two allocated treatment groups.

## Results

Of the 774 UK women included in follow up, 12 were lost to follow up and three died after discharge from hospital (Figure 1); data are available for 759 of the 771 surviving women (98%). Cause of death for the women who died was stroke (1); asthma (1); and non-Hodgkins lymphoma (1). Of the 827 children included in follow up, 12 were lost to follow up, 5 were excluded due to a problem arising at conception or embryogenesis and 19 died (Figure 2); data are available for 791 of the 803 surviving and normally formed children (98%). Of the 17 children who died before discharge from hospital, 13 were randomised before delivery and 4 after delivery. Four of these babies were stillborn and 13 died of prematurity. Cause of death for the two children who died after discharge from hospital was also prematurity.

Overall, 619 women completed their questionnaire and 659 families returned the children's questionnaire. Response was after a reminder for 359 families, and the questionnaire was completed by phone for an additional 47 families. Questionnaires were completed 24 months (median, inter quartile range 23–31 months) after the child was born (adjusted for gestation at birth up to 24 months). There was no difference in the response rate between the two allocated groups. In addition, there were no clear differences between women who responded to the questionnaire (n = 619) and those who did not (n = 140) in characteristics at trial entry, outcome at delivery or outcome at discharge following delivery (data not shown).

Of women included in the UK follow up, three quarters (74%) were primiparous, a quarter were less than 34 weeks gestation at trial entry, just over half (56%) had labour induced, two thirds (61%) were delivered by caesarean section and nearly half (46%) had severe pre-eclampsia (data not shown). For 132 children the family did not complete the questionnaire; but for two the families contacted us to say the child had severe disability and request that they have no further assessment. The remaining 130 children were no more likely to have developmental delay, reported by the general practitioner, than those children for whom the family completed a questionnaire (data not shown).

Overall for 688/759 (91%) women the general practitioner's questionnaire was completed. The majority (62%) responded after the initial questionnaire, some practices requiring reminders (34%). Fifty-one practices requested, and were sent, a copy of the trial consent form; of these 36 responded by returning the completed questionnaire. Payment was requested and provided for 36 practices (eight also requested a copy of the trial consent form). Five practices gave no response. No general practitioner could be identified for three women. The general practitioner's questionnaires were completed 23 months (median, inter quartile range 19–27 months) after the child was born (adjusted for gestation at birth up to 24 months). Based on information provided by the general practitioner we did not contact four families: the reasons were recent marital breakdown (2), recent death of husband (1) and drug addiction (1).

### Comparison of responses from general practitioners and families

Questionnaire responses were available from both general practitioners and women for 551/759 women (Table 1) and for 580/791 children (Table 2). For the women, responses were largely comparable although women reported fewer visits to the general practitioner, and being prescribed fewer drugs other than antihypertensives, than did general practitioners. This was despite the general practitioners completing the questionnaire slightly earlier than the women. For the children's questionnaires, again general practitioners reported more visits than did families; these data are not directly comparable, however, as families were only asked to report visits in the last six months

**Table 1:** Comparison of responses for the 551 women for whom there were questionnaires from both general practitioner and women

	**General practitioner**		**Women**	
	n = 551 (%)		n = 551 (%)	
*Visit/s to general practitioner*	531	(96)	411	(75)
Number of visits:	191		216	
1–5	161		75	
6–10	161		75	
> 10	168		77	
not known	11		43	
				
*Visits to hospital clinic*	222	(40)	199	(36)
Not known	6	(1)	6	(1)
				
*Admission to hospital*	45	(8)	89	(16)
				
Number of admissions to hospital/year*				
Reported at ≤ 24 months **				
Median {IQR}	0.63 {0.6–0.7}		0.5 {0.5–1.0}	
Range	0–2		0–4	
				
Reported at > 24 months ***				
Median {IQR}	0.4 {0.4–0.5}		0.4 {0.3–0.5}	
Range	0–1		0–2	
				
not known number of times admitted	7		10	
				
*Since Magpie pregnancy*				
Serious morbidity****	14	(3)	18	(3)
had antihypertensive since 6 weeks postpartum	126	(23)	107	(19)
*not known*	7		6	
				
Taking antihypertensive drug now	54	(10)	59	(11)
not known	6	-	2	-
				
Other prescribed drug/s for > 2 weeks	316	(57)	150	(27)
not known	6		6	-

* excludes admissions related to pregnancy

** general practitioner n = 335, women n = 166

*** general practitioner n = 216, women n = 385,

**** one or more of renal problems, stroke, severe hypertension

**Table 2:** Comparison of responses for 580 children for whom there were questionnaires from both general practitioner and family

	**General practitioner**		**Family**	
	n = 580 (%)		n = 580 (%)	
Visit/s to general practitioner				
*Since birth*	569	(98)		
*In 6 months preceding questionnaire*			295	(51)
				
Medication prescribed by general practitioner*	196	(34)	154	(27)
				
*Admission to hospital*	168	(30)	221	(38)
Reasons:				
*Respiratory infection*	54		56	
*Minor surgery*	35		38	
*Gastro-enteritis*	33		24	
Other	46		103	
				
***Number of admissions to hospital/year***				
Assessed at ≤ 24 months**				
Median {IQR}	0.7 {0.6–1.2}		0.5 {0.5–1.0}	
Range	1–3		1–3	
				
Assessed at > 24 months***				
Median {IQR}	0.4 {0.4–0.8}		0.4 {0.3–0.6}	
Range	1–4		1–4	
not known number of times admitted	28		2	

*medications (vitamin/mineral supplement, skin treatment, asthma treatment, gastro-intestinal/laxative, antibiotics)

** general practitioner n = 328, children n = 253

*** general practitioner n = 252, children n = 327

### Outcome for surviving women and children

#### Women

Overall serious morbidity potentially related to pre-eclampsia was reported for 31 (4%) women recruited in the UK; of whom 26 had severe hypertension (defined as taking two or more antihypertensives at time of completing questionnaire), six had renal problems, and one woman had had a stroke. Other morbidity was reported for 331 (44%) women, this included: minor ailments (colds, flu, diarrhoea and vomiting, backache) for 235 (31%) women, major psychiatric illness (psychosis or depression requiring treatment) for 144 (19%) and minor psychiatric illness (stress or anxiety not requiring treatment) for 49 (6%). Women's reporting of their fertility since the Magpie Trial and family history of pre-eclampsia and use of health care resources are in Table 3.

**Table 3:** Fertility, history of pre-eclampsia, and use of health care resources reported by the women

	**MgSO_4_**		**Placebo**	
	n = 303 (%)		n = 316 (%)	
**Contraception**				
Used since Magpie Trial	219	(72)	245	(78)
Not known	-	-	1	-
				
Decision about contraception influenced by pre-eclampsia	52	(17)	56	(18)
Not known	19	(6)	21	(7)
Reason given*:worried about having pre-eclampsia again	15		29	
advised not to take pill	22		14	
advised not to have/did not want more children	9		3	
not known	9		11	
				
**Fertility**				
Has **not **tried to get pregnant again	215	(71)	219	(69)
Not known	1		2	
Decision not to get pregnant influenced by pre-eclampsia	91	(30)	94	(30)
Not known	37	(12)	40	(13)
Reason given*:pre-eclampsia unpleasant experience	55		64	
did not enjoy pregnancy	11		8	
pre-eclampsia in previous pregnancy	8		7	
advised not to get pregnant	5		6	
would like to know more	3		3	
other	13		12	
not known	9		6	
				
Pregnancy since Magpie Trial	83	(27)	84	(27)
Pregnant now	24		23	
				
Has had pre-eclampsia again	13	(4)	14	(4)
Not known	25		26	
				
Family history of pre-eclampsia**	79	(26)	87	(28)
1^st ^degree relative	80		81	
2^nd ^degree relative	16		21	
not known	5		10	
				
***Help from other health care worker***	34	11	46	15
Health care worker	15		22	
Psychiatric services	14		11	
Alternative therapist	5		10	
Not known	-		3	

* some women gave more than 1 reason

** some women had more than one relative

#### Children

Over 30% of children were reported to have been admitted to hospital: chest infection and minor surgery being the most common reasons. Other morbidity was reported for 149 (26%) children, this included: minor ailments (allergic reactions, coughs, colds, ear infections). Prescribed medication for more than two weeks was reported for 126 (25%) children: these included vitamins (29%), asthma treatment (25%) and antibiotics (19%) (Table 2). There were no clear differences between the randomised groups in reported behaviour for the children (Table 4). The sole exception was children randomised magnesium sulphate were more likely to play happily with other children than those randomised placebo (RR 1.10 CI 1.02 to1.18). Of the 671 children with a completed questionnaire, the ASQ could be scored for 659 (98%).

**Table 4:** Reported behaviour for children with a completed questionnaire

	**Randomised before delivery**			
	**MgSO_4_** **n = 308 (%)**		***Placebo*** ***n = 301 (%)***	
Total responded to questionnaire	250	81%	254	84%

Does your child play happily alone for up to 10 minutes?				
Yes	210	(84)	201	(79)
Sometimes	33		40	
No	1		3	
not answered	6		10	
				
Is your child frightened in new situations?				
Yes	28	(11)	34	(14)
Sometimes	149		135	
No	66		70	
not answered	7		15	
				
Does your child play happily with other children if you are around?				
Yes*	220	(88)	204	(80)
Sometimes	22		34	
No	1		5	
not answered	7		11	
				
Does your child cling to you when you are with other people?				
Yes	29	(12)	35	(14)
Sometimes	127		126	
No	87		81	
not answered	7		12	
				
Does your child settle easily to sleep through the night?				
Yes	157	(63)	164	(65)
Sometimes	49		43	
No	36		36	
not answered	8		11	
				
No response to questionnaire	58	19%	47	16%

*MgSO4 versus placebo = RR 1.810 [CI 1.0210–1.182.94]

## Discussion

Data presented here provide further reassurance about the longer term safety of magnesium sulphate when used for women with pre-eclampsia. There appears to be no substantive effect on women's' subsequent fertility, or their use of health care services in the two years after the birth. As magnesium sulphate is associated with cerebral vasodilatation, and is a blocker of N-methyl-D-aspartate (NMDA) receptors in the brain, the pathway for anoxic cell damage, we hypothesized that *in utero *exposure might influence a child's development and behaviour. Data from the main Magpie Trial follow up study have already demonstrated that magnesium sulphate has no clear effect on the child's risk of severe neurodevelopmental delay [9]. These additional data from the UK provide further reassurance that *in utero *exposure to magnesium sulphate does not appear to have a major influence on behaviour at two years of age. The sole exception was that children of women randomised to magnesium sulphate were more likely to be reported as playing happily with others, than those of women randomised to placebo. Although this result is reassuring, it may reflect the play of chance and therefore should be interpreted with caution.

The high response rate to the postal questionnaires is a key strength of our study. By sending questionnaires to both the families and general practitioners we were able to achieve 98% follow up. For families we were unable to contact, or who did not respond to the questionnaire, information was provided by their general practitioner. Comparison of these responses is reassuring, in that they are largely comparable. This is consistent with the agreement between a parental questionnaire and paediatric assessment for children with a high prevalence of impairment [16]. The main differences between the two sources of information were that women and families reported fewer visits to the general practitioner, and women reported more visits to hospital and fewer prescribed drugs that were not antihypertensives. As visits to the general practitioner were so frequent, it is not surprising that families tended to underestimate the number. The higher reporting of hospital admission by women may reflect women counting attendance at a day unit as hospital admission, with general practitioners only counting over-night stays.

The follow up study did not begin until after recruitment closed, as this was when funding was secured. Nevertheless, use of the Office for National Statistics ensuring complete ascertainment of mortality, and accessing the National Health Service Tracing Scheme enabled us to trace all but four surviving mothers and their four children. An additional advantage of the separate system for ascertaining deaths was that unnecessary distress of contacting families if the child had died could be avoided, and if the mother died an appropriate approach could be made to the child's current carer.

Fewer families responded to the questionnaires than did the general practitioners. Our children's questionnaire asked for the child's current height and weight, information that was often missing from questionnaires returned. Some families told us they delayed returning questionnaires in order to take the child to a clinic to be weighed and measured. It is seems likely that this delay contributed to the non-response. As the data for height and weight were incomplete, they have not been reported. Our impression is that women and families largely valued being contacted for the follow up study. Only 12 families chose to opt out. Women returning the questionnaires, and being contacted for a home visit, often expressed their appreciation of our continued interest in their, and their child's, health and welfare. In addition, the questionnaire to women included three questions about their views of participation in the Magpie Trial, these data are reported elsewhere[14].

Data presented here provide further confirmation of the considerable morbidity experienced by women following childbirth. There is a growing awareness of the high level of health problems women experience, even after an uncomplicated pregnancy and childbirth [17]. Around 14% of women report health problems (such as back pain, exhaustion, anaemia, haemorrhoids, headaches and emotional difficulties) in the first eight weeks after the birth, and 10% in the subsequent 12–18 months[18] For women with pre-eclampsia, who are more likely to have had complications such as caesarean section or preterm birth, long term morbidity is probably higher[18]. In the Magpie Trial follow up study overall, only one third of women did not report any health problems, and even this may represent under reporting [10]. Women in the UK reported similar levels of health problems to women in other countries who participated in the Magpie Trial[10]. However, mental health problems were reported by a quarter of women in the UK, compared to just 6% of women overall in the follow up study [10]. This difference probably reflects substantial under ascertainment in low and middle income countries [10]. As our UK data are based on self reporting and information from general practitioners, the true level of mental health morbidity may be even higher; some women may not have recognised their symptoms or discussed them with their general practitioner. The high level of depression and anxiety after childbirth is important not only in its consequences for the woman, but also because it has been shown to be associated with impaired child development [19-22] Further understanding of how to prevent these adverse outcomes is required.

## Conclusion

Magnesium sulphate reduces the risk of eclampsia [8] without any substantive effect on longer term morbidity and mortality for the women or children [9,10]. Data presented here provide further reassurance about the longer term safety of magnesium sulphate when used for women with pre-eclampsia: there appears to be no influence on women's' fertility or her use of health care services, nor on the child's development or behaviour in early childhood. Sending postal questionnaires to families and general practitioners to assess the health of women and children is feasible, and can achieve a high response.

## Competing interests

The authors declare that they have no competing interests.

## Authors' contributions

RMDS wrote the first draft of the manuscript, with input from LD. All authors participated in reviewing and revising the manuscript and approved its final version.

## Pre-publication history

The pre-publication history for this paper can be accessed here:



## References

[B1] World Health Organization International Collaborative Study of Hypertensive Disorders of Pregnancy (1988). Geographic variation in the incidence of hypertension in pregnancy. Am J Obstet Gynecol.

[B2] Department of Health (1996). Confidential Enquiry into stillbirths and deaths in infancy.

[B3] Rosenberg K, Twaddle S (1990). Screening and surveillance of pregnancy hypertension – an economic approach to the use of daycare. Bailliere's Clin Obstetrics and Gynaecology.

[B4] Anthony J (1992). Improving antenatal care: the role of the antenatal assessment unit. Health Trends.

[B5] Bouvier-Colle MH, Salanave B, Ancel PY (1996). Obstetric patients treated in intensive care units and maternal mortality. European journal of Obstetric and Gynaecological Reproductive Biology.

[B6] Douglas KA, Redman CW (1994). Eclampsia in the United Kingdom. BMJ.

[B7] The National Institute for Clinical Excellence, The Scottish Executive Health Department, The Department of Health and Social Services and Public Safety Northern Ireland (2001). Why Mothers Die 1997–1999:the fifth report of the Confidential Enquiries into Maternal Deaths in the United Kingdom.

[B8] Magpie Trial Collaborative Group (2002). Do women with pre-eclampsia, and their babies, benefit from magnesium sulphate? The Magpie Trial: a randomised placebo-controlled trial. Lancet.

[B9] The Magpie Trial Follow-up Study Collaborative Group (2007). The Magpie Trial: a randomised trial comparing magnesium sulphate with placebo for pre-eclampsia. Outcome for children at 18 months. BJOG.

[B10] The Magpie Trial Follow-up Study Collaborative Group (2007). The Magpie Trial : a randomised trial comparing magnesium sulphate with placebo for pre-eclampsia. Outcome for women at two years. BJOG.

[B11] Magpie Trial Follow Up Study Collaborative Group (2004). The Magpie Trial Follow Up Study : outcome after discharge from hospital for women and children recruited to a trial comparing magnesium sulphate with placebo for pre-eclampsia [ISRCTN86938761]. BMC Pregnancy and Childbirth.

[B12] Farrell B, Duley L (2007). Doing the undoable: Magpie Trial long-tem follow-up. The Lancet.

[B13] Squires J, Potter L, Bricker D (1999). The ASQ user's guide for the Ages and Stages Questionnaires: a parent-completed, child monitoring system.

[B14] Smyth RMD, Duley L, Jacoby A, Elbourne D (2009). Women's experiences of participating in the Magpie Trial: a UK survey by postal questionnaire. Birth.

[B15] Bayley N (1993). Manual for the Bayley scales of infant development.

[B16] Fooks J, Mutch L, Yudkin P, Johnson A, Elbourne D Comparing two methods of follow up in a Multicentre randomised trial. Arch Dis Child.

[B17] Glazener C, Abdalla M, Stroud P (1995). Postnatal maternal morbidity: extent, causes, prevention and treatment. BJOG.

[B18] Brown S, Lumley J (2000). Physical health problems after childbirth and maternal depression at six to seven months postpartum. BJOG.

[B19] Murray L, Cooper PJ (1997). Effects of postnatal depression on infant development. Arch Dis Child.

[B20] Cooper PJ, Murray L (1998). Postnatal depression. BMJ.

[B21] Petterson SM, Burke Albers (2001). A Affects of poverty and maternal depression on early child development. Child Development.

[B22] Dawson G, Ashman S (2001). Preschool outcomes of children with depressed mothers. Child Development.

